# A Study on Market Segmentation According to Wellness Tourism Motivation and Differences in Behavior between the Groups—Focusing on Satisfaction, Behavioral Intention, and Flow

**DOI:** 10.3390/ijerph20021063

**Published:** 2023-01-06

**Authors:** Jun Lee, Jeong-Joon Kim

**Affiliations:** 1 Master of Tourism, Event, and Convention Management, Kyonggi University, Seoul 03746, Republic of Korea; 2 Department of Airline Services, Seowon University, Cheong-ju 28674, Republic of Korea

**Keywords:** wellness tourism, market segmentation, wellness motivation, satisfaction, behavioral intention, flow, wellness tourist type

## Abstract

The purpose of this study was to derive the visit motivations of wellness tourists and to derive strategies for the wellness tourism market through market segmentation based on visit motivations. First, this study derived seven motivators through a literature review with a discussion of experts: relaxation/healing/escape from everyday life, health improvement, novelty, luxury and prestige, self-examination/education, nature-friendly motivation, and social relations improvement. Then, in order to derive differentiated characteristics by motivation according to market segmentation, a difference analysis was conducted with the satisfaction, behavioral intention, and flow of wellness tourism participants. Data collection was carried out with the users of Chungcheongbuk-do wellness tourism products, and was supported by the Ministry of Culture, Sports, and Tourism (South Korea) from 2 September to 4 December 2021. Through the analysis in this study, it was first found that the wellness tourism motivations of wellness tourists were divided into a total of six factors (relaxation/healing/health improvement, novelty, luxury and prestige, self-examination/education, nature-friendly motivations, and social relation improvement). Then, as for market segmentation according to the visit motivations of wellness tourists, it was found that the markets were divided into “novelty-seeking type, comprehensive motivation-seeking type, neutral wellness-seeking type, and exploratory wellness-seeking type.” Finally, it was determined that there were significant differences in satisfaction, behavioral intention, and flow among those types of wellness tourist groups, and the average levels of satisfaction, behavioral intention, and flow were commonly lower in the neutral wellness-seeking type than in the comprehensive motivation-seeking type and the exploratory wellness-seeking type. This study derived the necessity to conceive differentiated strategies for the wellness tourism motivation group with the development of a wellness tourism motivation scale and a market segmentation study, and provided practical implications according to the characteristics of individual groups.

## 1. Introduction

Wellness tourism originated from the trend of wellness activities. The concept of wellness is very extensive and differs according to many studies, but it can be understood as balanced improvement activities not only for simple physical health promotion, but also for emotional/intellectual/social/mental/psychological/occupational/environmental factors [1]. As interest in wellness increases, the importance of wellness tourism, which links wellness with tourism activities, is also increasing in the tourism industry. In other words, wellness tourism has emerged as a kind of special interest tourism of people who travel in the pursuit of health and relaxation, which has to be investigated separately in tourism studies due to the fact that it is a characteristic distinct from general tourism [2]. As tourists’ interest in health increases due to COVID-19, wellness tourism considering health occupies an important part of tourism development in various countries, and academic interest in wellness tourism is steadily increasing [2,3,4,5,6].

As the demand for wellness tourism increases, as do its distinct characteristics, it becomes important to establish differentiated strategies with an analysis of the behavioral characteristics of wellness tourism participants in terms of efficient marketing of wellness tourism products and performance management [7]. That is, many analyses and studies are required to induce wellness tourists’ positive behavior and behavioral intentions, which is classified as a somewhat new type of tourism.

Considerable studies have been conducted in the field of tourism to increase tourists’ positive behaviors regarding tourism products, and many studies have mainly focused on the verification of the influencing relationships of variables (e.g., service quality, motivation) that affect behavioral intentions, e.g., [8,9,10,11,12,13]. Particularly, participation motivation has been verified as a variable that affects satisfaction and behavioral intentions, e.g., [14,15,16,17]. Likewise, satisfaction and behavioral intentions have been used together as dependent variables of motivation in many tourism and hospitality studies, e.g., [18,19].

Furthermore, studies related to flow and immersion have been conducted in tourism research in recent year, and flow experience has been widely used as a dependent or moderating variable to tourist behavior, e.g., [20,21,22]. Flow originating from positive psychology is a core component that can influence consumers’ positive behavioral intentions; thus, there is a need to apply it to tourism research [22,23]. Accordingly, it has been noted that wellness tourism research should be focused on studying consumer behavior related to satisfaction, behavioral intention, and flow, according to the motivation of wellness tourism.

However, studies related to motivations for participation in wellness tourism have not been able to derive concrete motivators regardless of the diversity of elements (beauty, food, meditation, exercise) due to the relatively short history of wellness tourism research. Thus, behavior studies based on motivation for wellness tourism have remained insufficient. Since wellness tourism’s motivators and product types are more diverse than those of other special interest types of tourisms, as proven in prior wellness research, e.g., [1,6], it is highly likely that the behavior of wellness tourism participants, which includes factors such as satisfaction, flow, and recommendation intentions, will appear differently depending on which factors of wellness tourism participation motivations are the main ones. In other words, motivations for participation in wellness tourism are not an influential variable that directly affects the behavior and behavioral intention of participants, and the degree of behavior appears differently according a certain motivator and its level. That is, it is judged that subdividing wellness tourism markets by main motivations for participation is more effective for studying wellness tourists’ behaviors at this point.

Therefore, in this study, (a) wellness tourism motive factors will be derived in detail, (b) the characteristics of each group will be derived from market segmentation according to wellness tourism motivators, and (c) differences in wellness tourism behavioral intentions and flow between the subdivided groups will be verified. Based on the results of this study, it is expected that along with concrete wellness tourism motivators, differentiated strategies for the operation of wellness tourism products will be derived through the characteristics of the segmented wellness tourism markets.

## 2. Literature Review

### 2.1. Concept of Wellness Tourism

Wellness is defined as the pursuit and action to maintain physical and mental health [24,25]. Many scholars and companies that emphasize the multiple dimensions of wellness, including GWI, have been emerging, and thus, various complex concepts of wellness centered on health have been created [2,26,27,28]. Despite this unclearness of the concept of wellness, tourism activities that focus on improving physical and mental health or discovering one’s ego and changing oneself have been referred to as wellness tourism, and wellness resorts, spas and meditation centers, etc., for wellness tourism have been appearing [27]. Since there are many interpretations of wellness, the concept of wellness tourism and its interpretations are diverse, but tourism that mainly aims at health and well-being is generally called wellness tourism. Romanova et al. [29] argued that wellness tourism is a sub-category of health tourism, and Stănciulescu et al. [30] suggested differences between health tourism and wellness tourism. Meanwhile, GWI [25] stated that wellness tourism and medical tourism show differences in concrete areas and purposes. Therefore, wellness tourism has been studied with many associations with healing, medical care, and health tourism. There are several definitions by scholars that are centered on the core elements of health, as shown in Table 1.

### 2.2. Wellness Tourism Market Segmentation and Motivation

Market segmentation is a strategy to divide a market with the same differentiated characteristics [35,36,37]. Market segmentation has mainly been studied based on geographical, demographic, psychological, and behavioral segmentation [36,38]. The main purpose of market segmentation is to secure competitive advantages by concentrating on marketing by group. In particular, market segmentation is a very important strategic tool in the service industry, which enables service providers to satisfy the needs of consumers more efficiently, and provides service providers with a broader view for the purpose of retaining existing customers and discovering new markets [39]. Understanding consumer needs, as well as efficient investments in and development of markets, are very important, as they lead to positive changes in consumer behavior, such as satisfaction, and ultimately lead to good marketing results for product and service providers [36].

Tourists and tourism markets can also be divided into groups with differentiated characteristics according to certain criteria. Even within the groups divided according to the types of tourism, there are further subdivided markets [40], and these subdivided tourist groups show differences in various aspects of behavioral characteristics. Therefore, product operation strategies should be established through an understanding of groups with similar characteristics [41,42]. As a result, market segmentation has been widely used in the tourism industry, such as in hotels, travel agencies, and tourist destinations [43].

Although there are a variety of criteria to classify tourist groups, tourism motivation is considered the most basic element in determining tourism. Consequently, tourism motivation is a variable frequently mentioned in market segmentation in the field of tourism science, and the importance of market segmentation through tourism motivations for efficient and suitable marketing strategies has been emphasized in many studies, e.g., [10,40,44,45,46].

Wellness tourism is a relatively new type of tourism, and along with the concrete derivation of wellness tourism motivators, in-depth studies on various factors affecting positive behavioral intentions, such as flow and satisfaction with wellness tourism participation programs, should be conducted. Several studies have analyzed wellness tourists’ motivations for participation, e.g., [31,47,48,49], and have verified the effects of their motivations on their improvement in areas such as satisfaction, revisit intentions, and loyalty, e.g., [31,48,50]. However, many studies conducted with wellness tourists have focused on the verification of factors affecting positive behavioral intentions rather than analyzing differences in a variety of behavioral characteristics with differentiated groups according to certain criteria. Therefore, studies that analyzed differences in behavioral characteristics between market groups divided according to wellness tourism motivations are relatively few.

Wellness tourism has been attracting more attention recently due to COVID-19, and it is now more urgent to establish differentiated strategies by deriving complex motivators and analyzing differences in behavior by group through group segmentation according to the motivations. Therefore, this study raises the necessity of an analysis of differences in behavioral characteristics between groups according to market segmentation by motivation, which is important in the operation and management of differentiated wellness tourism program products.

### 2.3. Derivation of Wellness Tourism Motivators

Motivation is a sort of process that causes people to act [10]. Motivations are driven by human desires, including internal and external forces [51]. Motivations are the most important variable for understanding consumer behavior [52,53]. In tourism studies, tourism motivations have been extensively studied based on push–pull factors [53,54,55,56]. Motivations for wellness tourism have also been studied frequently, and most scholars have considered motivations based on items related to health and healing [27,31,57]. Other complex motivations show differences, as shown in Table 2.

As mentioned earlier, since consumers have different needs and behavioral characteristics, they should be divided into groups and accurately analyzed if efficient marketing strategies were to be implemented by companies [6]. In tourism studies, market segmentation studies have been carried out in relation to tourism consumers based on a variety of variables. Among them, many studies have segmented the market based on tourism motivations [46,53,58]. However, not many studies in the field of wellness tourism have segmented the market based on motivators thus far. Damijanić [46] conducted cluster analysis based on wellness tourism motivators and classified wellness into Cluster 1: immaterial wellness, Cluster 2: high wellness, and Cluster 3: low wellness. Many other studies have looked at wellness tourism motivators, but did not segment the market using wellness tourism motivators. Since the wellness tourism market is composed of consumers with various motivations, as shown in Table 2, the necessity of market segmentation to compare individual behavioral characteristics is greater.

Based on the above studies, motivations for participation in wellness tourism were re-divided into seven dimensions after discussion by three experts in the academic field whose majors and academic interests were related to wellness tourism. The seven dimensions were composed of a total of twenty-nine items, including “relaxation/healing/escape from daily life,” with four items; “health improvement,” with six items; “novelty,” with three items; “luxury and prestige,” with four items; “self-examination/education,” with four items; “nature friendliness,” with four items; and “social relations improvement,” with four items. Contents related to the foregoing are shown in Table 3.

### 2.4. Satisfaction and Behavioral Intention

Satisfaction is determined by the difference between the consumer’s expectations before purchase and their actual perception after purchase [61,62]. Customer satisfaction has been regarded as an important variable in consumer behavior studies, and is an evaluation of the consumer’s experience of products and services [63]. Satisfaction has mainly been used as an independent variable in consumer behavior studies and has been studied along with behavioral intention and influence, e.g., [16,63,64,65,66]. Huang et al. [62] studied the effects of tour guide intervention on tourist satisfaction and behavioral intention, and Chan et al. [67] demonstrated the effects of service performance on tourists’ satisfaction and behavioral intentions. Satisfaction has been treated as a major study variable related to behavioral intention in tourism consumer behavior studies. In this study, it denotes to what degree wellness tourists were satisfied with wellness tourism products, and is composed of a single factor to measure overall satisfaction with wellness tourism products.

Behavioral intention refers to the likely behavior expected by consumers in the future [68]. Behavioral intention has been studied with various concepts and methods [67], and the measurement variables are evolved and used centered on the scale developed by Zeithaml et al. (1996) and Baker and Crompton (2000) [67,68]. The 13 variables of behavioral intention could be largely divided into loyalty, replacement tendency, payment intention, and response to problems [68]. Tourism studies mainly look at loyalty, revisit intention, and positive word of mouth (recommendation intention) as behavioral intentions of tourists. For example, Bayih and Singh [16] composed tourists’ behavioral intentions with revisit and recommendation intentions. Kim, Ritchie, and Tung [69] set revisit intention, recommendation intention, and re-practice as components of behavioral intention. In addition, there are scholars who included more extensive components, such as loyalty and word of mouth intention, in their studies on behavioral intention, e.g., [62,70,71]. That is, tourists’ behavioral intention can be defined as tourists’ intention for behavior that will occur in the future after visiting/experiencing tourism products and tourist destinations. In this study, the behavioral intention of wellness tourists was composed of two factors, revisit intention and recommendation intention, referring to studies conducted by [16,69,70] and considering the emergence of the wellness tourism market.

### 2.5. Wellness Tourism Flow

Recognizing the importance of tourist flow in tourism studies, many studies have been carried out, e.g., [21,23,72,73]. Flow originates from flow experience, which is a psychological concept, and means a state where an individual fully concentrates on his or her activities or actions and feels the optimal experience [74]. Kim and Thapa [21] defined this as a state where a tourist is engaged in tourism activities or companions while experiencing tourism.

In addition, flow state is sometimes used interchangeably with immersion. This refers to a mental state or feeling where a consumer is immersed in the current experience and forgets his/her ego and external things [29,75,76], which is consistent with the tourist’s flow status dealt with in this study. However, since immersion constitutes breaking away from the external world to become immersed in a new world, it is widely used in relation to virtual reality in the e-service industry [77,78]. Therefore, the factor of how much tourists flow in tourism experiences will be called flow.

An increase in flow has been widely used as an independent variable in consumer behavior studies, and has been verified to affect consumer behavior outcome variables such as satisfaction [21,76,79]. Due to the influencing relationship, tourists tend to ask for flow experiences more often when they visit tourist destinations. As a result, tourism product providers are paying attention to this, as it is relevant [80].

In summary, the concept of wellness tourism flow can be defined herein as the degree to which wellness tourists intensively participate in wellness tourism products. Since the terms engagement and flow are used in the field of wellness tourism studies, some studies on wellness tourist flow behavior, which is dealt with in this study, have been performed using these terms, e.g., [48,81,82]. Nevertheless, flow studies conducted with wellness tourists are still quite insufficient. In this study, wellness tourism flow was composed of a single factor, referring to previous studies on flow in the field of tourism using flow and immersion. These studies indicated the state of tourists who flowed into a tourism experience, e.g., [21,73,80].

## 3. Methodology

### 3.1. Research Instrument

For the analysis of market segmentation according to wellness tourism motivations and differences in behavioral characteristics between groups, which is the purpose of this study, questionnaire measurement items were composed as shown in Table 4. They consist of 6 sub-categories, and the total number of items is 54. Except for the demographic questionnaire survey, all items were set based on 1 point (not at all) to 7 points (very much) using the Likert 7-point scale to increase the accuracy of the results.

### 3.2. Data Collection

This study conducted a questionnaire survey on wellness tourism product participants in Chungju city, a wellness tourism cluster area designated by the Ministry of Culture, Sports, and Tourism of the Republic of Korea. Specifically, the survey was given to participants in seven wellness tourism products that ran from 27 August to 5 December 2021 (“Wellness Body & Mental Rest Sleep Premium,” “Wellness One Day Singing Ball Meditation Trip,” “Wellness One Day Healing Trip,” “Wellness Happy Family Mind Trip,” “Wellness Stay,” “Wellness Alley Stay,” “Wellness Pause”) at the Godowon Meditation Healing Center in Chungju established by the Morning Letter Cultural Foundation. The direct survey method was used for all field participants, and a paper questionnaire and a mobile survey method were used in parallel. The survey period was approximately 3 months, and 266 copies of final valid samples were used.

### 3.3. Data Analysis

This study used the SPSS 26.0 program for questionnaire analysis. First, an exploratory factor analysis (EFA) was conducted to analyze the reliability and validity of each item, and the reliability of each variable was analyzed. This study used the Varimax method for the rotation of the factors, and the number of factors was analyzed using a set with an eigenvalue of 1.0. Second, a non-hierarchical/hierarchical cluster analysis method was used to segment the market by deriving group characteristics with wellness tourism motivations. Cluster analysis is a technique that divides a sample into many groups by verifying homogeneity based on sub-characteristics [83]. Finally, to analyze differences in satisfaction, revisit intention, recommendation intention, and flow by group, a one-way ANOVA analysis was performed. Ex post analysis was carried out to derive detailed differences when the results of one-way ANOVA indicated that there were differences among the four groups. Among various ex post analysis methods, Scheffe’s ex post analysis was performed to analyze differences between the groups.

## 4. Result

### 4.1. Demographic Analysis

A main characteristic of the demographic questionnaire survey results is that there was a relatively higher proportion of women than men. In addition, the analysis indicated that there were many elderly people among wellness tourists, and the participants’ incomes were relatively high. In addition, it was found that many the participants had diseases. Details of the demographic questionnaire survey results are shown in Table 5.

### 4.2. Reliability and Validity Test

#### 4.2.1. Wellness Tourism Motivation

In this study, exploratory factor analysis was performed first to refine the initial items of the wellness tourism motivation scale derived earlier, as well as to verify their suitability.

As a result of exploratory factor analysis in the first round, which was performed using SPSS 26.0, the first of the items in the “relaxation/healing/escape from daily life” category was deleted due to its lower factor loading value. On the second round of EFA, the suitability and reliability of the motivation scale model were verified as appropriate. However, the factors “relaxation/healing/escape from daily life” and “health improvement” that were initially set were analyzed as one factor.

Based on the standard value of the factor loading value, 0.4, which is the standard factor loading value asserted by Hinkin [84] and Howard [85], the loads of all factors were confirmed to be appropriate. Eigenvalues, variance explanatory power, and reliability of each factor were also shown to be appropriate. The KMO value was at least 0.7, and Bartlett’s sphericity test value was at least 0.5, indicating that the fit and correlations between the factors were quite sufficient [86]. The EFA result of wellness tourism motivators is shown in Table 6.

Accordingly, the wellness tourism motivation scale developed in this study was finally composed of “relaxation/healing/health improvement,” “self-examination/education,” “nature friendliness,” “luxury/prestige,” “social relations improvement,” and “novelty,” as two of the variables presented earlier were merged.

#### 4.2.2. Satisfaction

As a result of factor analysis of measurement items regarding overall satisfaction with wellness tourism products, the reliability and validity of all measurement items were identified as suitable at a high level. Based on the criteria applied in the process of development of the wellness tourism motivation scale, the total variance explanatory power was identified as 90.633%, the KMO value as 0.764, the Bartlett sphericity test value as 780.175, and the factor loading as at least 0.5, indicating that the factor analysis model was valid. Cronbach’s á value was also found to be very high, at least 0.9; thus, the suitability of the overall satisfaction measurement variable used in this study was verified. The detailed EFA result of satisfaction is illustrated in Table 7.

#### 4.2.3. Behavioral Intention

As a result of factor analysis for the wellness tourism product behavioral intention measurement items, the reliability and validity of all measurement items were identified as highly appropriate. First, in the case of revisit intention, the total variance explanatory power was 86.985%, the KMO value 0.748, the Bartlett sphericity test value 630.693 ***, and the factor loading at least 0.5, indicating that this factor analysis model was valid. Cronbach’s á value was also shown to be very high, at least 0.9; thus, the suitability of the items was verified. Table 8 shows the relevant results.

In the case of recommendation intention, the total variance explanatory power was 94.774%, the KMO value 0.744, the Bartlett sphericity test value 1221.156 ***, and the factor loading at least 0.5. Cronbach’s á value was also shown to be very high, at least 0.9; thus, the suitability was verified. Table 9 shows the relevant results.

#### 4.2.4. Flow

Exploratory factor analysis was conducted for flow, and according to the results, the total variance explanatory power was 87.206%, the KMO value was 0.735, the Bartlett sphericity test value was 651.647 ***, and the factor loading was at least 0.5, indicating that this factor analysis model was valid. Cronbach’s á value was also shown to be very high, at least 0.9; thus, the suitability of the flow measurement items used in this study was verified. The results are shown in Table 10.

### 4.3. Market Segmentation according to Wellness Tourism Motivations (Cluster Analysis)

This study conducted cluster analysis for market segmentation according to wellness tourism motivations using the SPSS 26.0. In the cluster analysis method, hierarchical cluster analysis is performed first to determine a good number of groups to create through the division of markets, and K-means cluster analysis is then performed to determine the optimal number of clusters [87,88,89].

Since Ward’s method is most suitably used for hierarchical cluster analysis [83], this study carried out hierarchical cluster analysis with this method first. Referring to the dendrogram visualization results of cluster analysis, it was judged appropriate, after expert discussion, to divide the wellness tourism market into four groups, and so the number of clusters was set to four to conduct k-means cluster analysis. As a result of one-way ANOVA, significant differences between the four groups were found in all factors of motivation, with an appropriate F-value (0.000). Therefore, wellness tourism product participants were finally divided into four clusters, and through expert discussion and analysis of each characteristic, Cluster 1 was called a novelty-seeking type, Cluster 2 a comprehensive motivation-seeking type, Cluster 3 a neutral wellness-seeking type, and Cluster 4 an exploratory wellness-seeking type. The detailed results are shown in Table 11.

#### 4.3.1. Novelty-Seeking Type (Cluster 1)

The samples belonging to cluster 1 were found to be a group with a high desire to seek the novelty of wellness tourism. In particular, cluster 1 showed characteristics that indicated that nature friendliness and novelty were overwhelmingly higher than other visit motivations. This cluster can be regarded to be a group that recognizes wellness tourism as a new form of tourism rather than having a high overall understanding of wellness tourism; this group wants to enjoy the benefits of the universally known nature friendliness. That is, it can be judged that most tourists are not familiar or vaguely familiar with wellness tourism, and are highly likely to have experienced wellness products for the first time. Therefore, cluster 1 was called wellness tourism’s “novelty-seeking type”.

#### 4.3.2. Comprehensive Motivation-Seeking Type (Cluster 2)

Cluster 2 was shown to be a group with a very high level of motivation for wellness tourism. As shown in Table 11, Cluster 2 had very high scores, no lower than 5.7, for all wellness tourism motivation items. Consequently, Cluster 2 was judged to be a group with a very high overall understanding of wellness tourism. In addition, a characteristic that was found to be different from other groups is that Cluster 2 had a very high average in the visit motivations “luxury/prestige” and “social relations improvement.” That is, Cluster 2 is highly likely to be a group that has encountered or experienced wellness tourism extensively, and is interpreted to be a high-level tourist group that hopes to equally enjoy the many benefits and effects that can be obtained from it. To reflect the characteristics of a group that wants the overall benefits of wellness tourism rather than certain motivations, Cluster 2 was called “comprehensive motivation-seeking type”.

#### 4.3.3. Neutral Wellness-Seeking Type (Cluster 3)

Similar to Cluster 2, Cluster 3 showed uniform measurement scores for visit motivations in general rather than preferring certain motivations. However, unlike Cluster 2, the scores were generally low. Therefore, Cluster 3 was interpreted to be a group with an average level of demand for all wellness tourism motivators. Apart from “nature friendly,” which was high in all groups, the average score of the motivator “relaxation/healing/health improvement” was relatively high compared to other motivators. Cluster 3 can be seen as a group with a high demand for the experience of basic functions of wellness tourism. In this study, Cluster 3 was regarded as a group in the stage of gradually increasing their level of understanding of wellness tourism, and was called the “neutral wellness-seeking type” to reflect the characteristics above.

#### 4.3.4. Exploratory Wellness-Seeking Type (Cluster 4)

Cluster 4 is a group that had relatively low scores in “social relations improvement” and generally high average levels for other wellness tourism motivations. This group had higher scores of the motivators “novelty, self-examination and education, relaxation/healing/health improvement” compared to other groups, thereby showing a relatively active attitude toward wellness tourism experience functions, and thus it is regarded as a group with inquiring/exploratory attitudes. That is, Cluster 4 is a group that mainly aims at self-development and reflection by experiencing and learning new things through wellness tourism. Therefore, cluster 4 was called the “exploratory wellness-seeking type”.

### 4.4. Analysis of Differences in Behavioral Characteristics by Motivation Group

This study was designed to analyze differences in the behavioral characteristics among the four groups divided by motivation. Therefore, one-way ANOVA was performed with certain specific factors (behavioral intention and flow) previously selected as important factors.

To that end, a null hypothesis (H0) was set as “There is no difference in the means among the four groups” and an alternative hypothesis (H1) was set as “There is a difference in the means of the four groups” to test whether there were differences in the means of certain variables by group. As for the process of analysis of differences in behavioral characteristics, ANOVA was carried out, followed by Scheffe’s post hoc analysis. The the F value and the significance probability value (*p* < 0.05) were checked between the groups, in order of precedence, in the cases where ANOVA indicated that there were differences among the four groups. ANOVA revealed whether the differences were statistically significant, but was limited regarding the ability to inform what kinds of differences exist between which groups. Consequently, the post hoc analysis was performed for that information.

Therefore, differences in the means of satisfaction, behavioral intention (revisit intention, recommendation intention), and flow were analyzed among the four groups. As a result, it was verified that the groups showed differences in the means of all four variables, as shown in Table 12. Therefore, it was decided that Scheffe’s post hoc analysis would be performed.

#### 4.4.1. Differences in Satisfaction by Wellness Tourism Product Motivation Group

The analysis conducted earlier indicated that there were differences (t = 5.548, *p* < 0.01) in satisfaction with wellness tourism products among the wellness tourism product participant groups. Therefore, Scheffe’s post-tests were carried out, and the results indicated that there were significant differences in overall satisfaction with wellness tourism products between the comprehensive motivation-seeking group and the neutral wellness-seeking group, as well as between the neutral wellness-seeking group and the exploratory wellness-seeking group. The mean of overall satisfaction of the overall motivation-seeking group was approximately 0.481 (*p* < 0.01) higher than that of the neutral wellness-seeking group, indicating that the overall motivation-seeking group was more satisfied with the wellness tourism products than the neutral wellness-seeking group. On the other hand, the mean of overall satisfaction of the exploratory wellness-seeking group was approximately 0.503 (*p* < 0.05) higher than that of the neutral wellness-seeking group. The detailed results are shown in Table 13.

#### 4.4.2. Differences in Behavioral Intention by Wellness Tourism Product Motivation Group

The analysis performed earlier indicated that there were significant differences (t = 6.763, *p* = 0.000) in intention to revisit wellness tourism products among the participant groups. In particular, the results of Scheffe’s post hoc tests indicated that there were significant differences in wellness tourism product revisit intention between the comprehensive motivation-seeking group and the neutral wellness-seeking group, as well as between the neutral wellness-seeking group and the exploratory wellness-seeking group. In detail, the mean of intention to revisit of the overall motivation-seeking group was approximately 0.574 (*p* = 0.000) higher than that of the neutral wellness-seeking group. In addition, the mean of revisit intention of the exploratory wellness-seeking group was approximately 0.537 (*p* < 0.01) higher than that of the neutral wellness-seeking group. The detailed results are shown in Table 14.

The analysis conducted earlier indicated that there were significant differences (t = 7.067, *p* = 0.000) in intention to recommend wellness tourism products among the participant groups. In particular, the results of Scheffe’s post hoc tests indicated that there were significant differences in wellness tourism quality factors between the comprehensive motivation-seeking group and the neutral wellness-seeking group, as well as between the neutral wellness-seeking group and the exploratory wellness-seeking group. In detail, the mean of intention to recommend of the overall motivation-seeking group was approximately 0.637 (*p* = 0.000) higher than that of the neutral wellness-seeking group. In addition, the mean of recommendation intention of the exploratory wellness-seeking group was approximately 0.588 (*p* < 0.01) higher than that of the neutral wellness-seeking group. The detailed results are shown in Table 15.

#### 4.4.3. Differences in Flow by Wellness Tourism Product Motivation Group

The analysis carried out earlier indicated that there were significant differences (t = 13.168, *p* = 0.000) in flow among the participant groups. The results of Scheffe’s post hoc tests indicated that there were significant differences in wellness tourism quality factors between the comprehensive motivation-seeking group and the neutral wellness-seeking group, as well as between the neutral wellness-seeking group and the exploratory wellness-seeking group. In detail, the mean of flow of the overall motivation-seeking group was approximately 0.946 (*p* = 0.000) higher than that of the neutral wellness-seeking group. In addition, the mean of flow of the exploratory wellness-seeking group was approximately 0.834 (*p* < 0.01) higher than that of the neutral wellness-seeking group. The detailed results are shown in Table 16.

## 5. Conclusions and Implications

The interest in and importance of wellness tourism are gradually increasing in various cases, such as well-being and the desire for personal health promotion due to COVID-19. However, studies on the behavior of wellness tourists are quite insufficient. Wellness tourism not only has the simple function of healing and cure, but also has many efficacies [25]. This implies that wellness tourists also have different motivations for wellness tourism. These motivations have been the main criteria for market segmentation, and intensively managing tourists through market segmentation is very important in the operation of tourist destinations and tourism products. Therefore, this study was intended to develop a wellness tourism visit motivation scale and segment wellness tourists by market in order to study the differences in behavioral characteristics between the groups.

### 5.1. Summary of Results

In this study, a wellness tourism motivation scale was developed in detail. A draft version was completed through expert discussion based on previous studies, and based on this, an EFA was conducted with a participant survey. As a result, among the seven motivators initially set, “relaxation/healing/escape from daily life” and “health improvement” were combined into one factor so that six dimensions (“relaxation/healing/health improvement,” “novelty,” “luxury and prestige,” “self-examination/education,” “nature friendliness,” and “social relations improvement”) were finally derived.

Based on the derived visit motivations, this study segmented the wellness tourism participants by market, and through the processes of hierarchical and non-hierarchical cluster analyses, the segmented participants were finally described as four groups (novelty-seeking type, comprehensive motivation-seeking type, neutral wellness-seeking type, and exploratory wellness-seeking type). The result indicating that visit motivation scores were the highest for nature friendliness in all four groups meant that the brand image of tourism emphasizing feeling nature is embedded in the public’s perception of wellness. Apart from nature friendliness, the four groups each showed different characteristics. This is similar to the wellness tourism market segmentation by motivation studied by Damijanić [39], but a difference exists in that this study classified wellness tourism participants into four groups. The results of wellness tourism market segmentation suggest that analysis of various markets is also important for the efficient operation and marketing of tourist destinations and tourism products in the wellness tourism industry.

Significant differences appeared regarding satisfaction with wellness tourism products, revisit intention, recommendation intention, and flow by group, and a post hoc test was carried out for the differences. According to the results, differences appeared in all groups except for the novelty-seeking type (comprehensive motivation-seeking type ≠ neutral wellness-seeking type, neutral wellness-seeking type ≠ exploratory wellness-seeking type). The neutral wellness-seeking group commonly showed lower means in all variables (satisfaction, revisit intention, recommendation intention, and flow) than the comprehensive motivation-seeking group and the exploratory wellness-seeking group. Since the neutral wellness-seeking group showed the lowest scores in satisfaction, behavioral intention, and flow related to wellness tourism, despite the fact that the level of demand for motivations for participation in wellness tourism of this group was not low, this group is interpreted to be the most cold-hearted and demanding group among the participating groups. It is also considered that the scores for the behavioral characteristics in relation to wellness tourism products of this group are low because this group is gradually increasing its basic understanding of wellness tourism, and does not have much desire for any certain motivation. On the other hand, the reason why the overall satisfaction of the comprehensive motivation-seeking type and the exploratory wellness-seeking type is high is interpreted to be the fact that there is a high level of understanding of various aspects of wellness tourism, because these groups have the characteristic of expecting high-level exploratory and self-examination beyond the basic functions of wellness tourism, thus leading to diverse levels of satisfaction. The foregoing means that the overall program operation of the current wellness tourism products in South Korea has not only basic functions, but also exploration and self-examination functions.

### 5.2. Theoretical Contributions

Based on the results of this study, theoretical implications reflecting the characteristics of each group will be provided. First, this study developed a wellness tourism motivation scale by deriving and verifying wellness tourists’ tourism motivations in detail. This scale can present predisposing factors for many studies analyzing the visit motivations of wellness tourists in the future. In addition, the tourism motivators indicated that wellness tourists visit wellness tourist destinations or participate in products not only for the purpose of simple healing/nature friendliness, but also to reap a variety of benefits. As with the previous studies, e.g., [31,46,60], this suggests that studies on the visit motivations for and efficacy of wellness tourism should be conducted from more diverse perspectives. These findings suggest that previous studies on the healing/cure-centered effects of wellness tourism should be focused on a wider range of benefits and motivations of wellness tourism destinations and products.

Second, this study showed that wellness tourism consumers are divided into various groups by providing differences between wellness tourism groups through wellness tourism market segmentation, according to participation motivations. This is consistent with Zhang and Marucssen’s [40] argument that the types of tourism are further subdivided into multiple markets. The results of this study emphasize, once again, the importance of dividing the wellness tourism market into various groups rather than looking at it as one in studies of consumer behavior in the wellness industry, as prior studies have emphasized, e.g., [40,45,46]. Concretely, since behavioral characteristics show some differences by group, the results of this study suggest that effective marketing and operation of wellness tourism products are possible only when differentiated strategies are conceived by a group. All four clusters showed significant differences and characteristics in relation to specific motivators, and the three groups, except for the novelty-seeking group, also showed differences regarding some tourists’ behaviors and behavioral intentions. Thus, there is a significant need for in-depth study of each group, and the various interpretations of the results should be applied to the operation of wellness tourism products.

### 5.3. Practical Contributions

Practical implications will be provided based on the results of this study. Concretely, the results suggest that wellness tourist destinations and product operators must conduct strategic segmented market management in consideration of the characteristics of each group.

First, attention should be paid to the “neutral wellness-seeking group.” Through colligation of the results, this study deduced the fact that the neutral wellness-seeking group was the most sensitive group of the participants. In particular, this group showed the lowest scores in behavior (satisfaction, recommendation intention, revisit intention, flow) despite having scores that were generally not low for various visit motivators. Consequently, efforts to prevent the sharing of negative opinions on wellness tourism products should be made by implementing intensive management strategies, such as receiving complaints and improving them with periodic surveys for the relevant group. In addition, in the case of the neutral wellness-seeking group, since the score of the “healing and relaxation” motivation was relatively high, although the means of the visit motivation scores were generally plain, it is necessary to operate a program that can maximize the effect of escape from daily life.

Next, the “novelty-seeking group” has a high tendency to recognize and experience wellness tourism as a new type of tourism, because this group consists of participants in the early introductory stage with relatively low overall understanding of wellness tourism. Therefore, rather than overly focusing on the wellness program per se, it is required to establish strategies to gradually increase loyalty to wellness tourism by expanding pleasures (recreational elements) associated with participation in wellness tourism. It is judged that the addition of recreational elements to the program, such as linkages with nearby tourist destinations, will be helpful, and it is necessary to continuously emphasize the reinforcement of programs related to natural elements and the professionalism of manpower.

The “comprehensive motivation-seeking group,” which had high-level means for all motivations for participation in wellness tourism, had a high degree of understanding of wellness tourism were clearly ready to enjoy the many benefits to be obtained from participating in wellness programs. Therefore, the diversification of wellness programs was judged to be helpful for continuous participation. Furthermore, if the members of this group become loyal customers, the expansion of programs operating with a small number of participants, the provision of personalized wellness programs that concretely reflect individuals’ needs, and products in combination with luxury tourism can ensure continuous visits by this group.

Next, the “exploratory wellness-seeking group” had an overall high level of means for all wellness tourism motivations, next to the comprehensive motivation-seeking group, and had higher means for the “novelty, self-examination and education, relaxation/healing/health improvement” motivators compared to other groups, thereby showing relatively active and inquiring/exploratory attitudes toward the wellness tourism experience function. Thus, educational contents that will enable inquiring about wellness tourism and nature should be concretely provided in the wellness tourism program. In addition, this group had the highest mean for satisfaction, and generally high means for revisit intention, recommendation intention, and flow. Thus, these participants can be a sort of effective marketing means that can positively promote wellness tourism programs to acquaintances. To that end, strengthened customer management measures such as continuous provision of information on wellness tourist destinations or products, promotions, and event benefits should be devised. However, since this group had relatively low levels for the “social relations improvement” motivator compared to other groups, care must be taken, because excessive provision of programs to enhance interaction between participants or program operators may lead to increased rejection of products and negative effects.

Lastly, strategies should also be supplemented according to the common characteristics of the market segments. It can be seen that the motivator of “nature friendliness,” which had the highest scores in all groups among various visit motivations, should be essentially included in wellness tourist attractions and products. Furthermore, the relaxation/healing/health improvement motivator also showed relatively modest differences between clusters rather than other motivators; thus, this indicates that general participants want the basic healing and cure benefits of wellness tourism, as proven by previous studies, e.g., [4,5]. Therefore, differentiated tourist destinations and product marketing methods should be carried out according to individual groups centered on these common motivations of nature and healing. For instance, if the natural environment around wellness facilities is linked well and the physical environment is also improved as nature-friendly, there is a high possibility of inducing visitors’ positive behavior towards wellness tourism.

### 5.4. Limitations and Future Research Agenda

Although this study provides several implications, it has some limitations. First, the survey was conducted only with wellness tourism product participants in a certain destination. Since representative themes vary by wellness tourism region and product, motivations for visiting wellness tourism products may differ. In the future, it will be necessary to conduct studies with more diverse wellness tourist destinations or wellness tourism products, and to compare them according to destination. Next, the variable behavioral intention was composed of only revisit intention and recommendation intention. If various factors for behavioral intention were constructed and studied, more implications could be drawn in relation to the comparison of the behavioral characteristics of the participant market. This suggests a need for future studies. Lastly, with regard to differences between groups, they were found only between two particular groups (comprehensive motivation-seeking type and neutral wellness-seeking type, exploratory wellness and seeking type-neutral wellness-seeking type), not between different groups. This comes from the result in which the neutral wellness-seeking group showed a low mean score in the overall characteristics. This is thought to be a limitation derived from the limits of study subjects regarding the wellness tourism products mentioned above. Therefore, it would be beneficial if differences between more diverse groups could be derived in future studies.

## Figures and Tables

**Table 1:** Previous studies on wellness tourism.

Authors	Definition of Wellness Tourism
Lim et al. [31]	Everything related to travel that mainly aims at maintaining and promoting health.
Korea Ministry of Culture, Sports and Tourism [32]	A new trend in tourism that promotes health and ultimately improves the quality of life through tourism activities such as enjoying spa, recreation, beauty, and health care.
Korea Tourism Organization [33]	A series of tourism activities based on the premise of travel according to the tourism motivations and purposes in pursuit of wellness, and facilities, activities, and programs that enable enjoying these activities must be included.
Voigt [34]	Tourism that includes the elements of and efficacy for health and wellness.
Stănciulescu et al. [30]	Tourism for the purpose of maintaining physical and mental balance, such as the pursuit of self-esteem, beauty, relaxation, and physical health.
GWI [25]	Tourism, such as the desire to maintain or promote personal well-being.

**Table 2:** Prior studies of motivation of wellness tourism.

Motivating Factors	Type of Wellness Destination	Ref.
Relaxation, self-exploration, accessibility, novelty, sightseeing, convenience for touring, accessibility	Arboretum	Lim et al. [31]
Health trend, relaxation and reward, novelty, cultural and natural heritage, entertainment and recreation, landscape	Wellness hotels	Damijanić [46]
Recreation, relaxation, mental therapy, enhancement of quality of life, effortless activity, health consciousness, experiencing nature, physical therapy, social activity, meditation, learning new things, curiosity, shopping health products		Blešić et al. [52]
Tourist destination, relaxation, local people, culture, nature	Resorts	Damijanić and Šergo [45]
Relaxation and relief, health and beauty, escape, self-development, travel motivation	Spa and wellness tourism destination	Hashim et al. [57]
Seeking spirituality, enhancing mental well-being, enhancing physical condition, controlling negative emotions	Yoga tourism destination	Lehto, Brown, Chen, and Morrision [59]
Prestige and luxury, novelty and knowledge, self-development, relaxation and escape	Wellness tourism destination	Kim et al. [48]
Medical/cosmetic, corporeal/physical, escapism/relaxation, hedonistic/experiential, existential/psychological, spiritual, and community-oriented		Smith and Kelly [27]
(Push) Mental and physical exhaustion, stress, loneliness, obsession, loss of religion, addiction to technology, not enough time spent outside/(Pull) Healthy, happiness, self-esteem, beauty, self-development, rehabilitation, simple life		Smith and Puckzo [60]
Movement and fitness, healthy food and diet, meditation and mindfulness, rest and relaxation, learning about wellness, self-care, and nature and disconnect		Kessler et al. [49]

**Table 3:** Motivations for participation in wellness tourism.

Dimensions	Items of Prior Studies
Relaxation/healing/escape from daily life	Relaxation, relief, escape, rehabilitation, happiness, meditation, controlling negative emotions, mindfulness, hedonism, enhancing of quality of life
Health improvement (mental and physical)	Health, beauty, enhancing mental well-being, enhancing physical condition, medical/cosmetic, shopping health products, corporeal and spiritual purpose
Novelty	Novelty, curiosity, travel, tourist destination, sightseeing, local culture
Luxury and prestige	Prestige, luxury
Self-examination/education	Self-development, self-esteem, self-care, learning about wellness/new things, knowledge
Nature-friendliness	Nature and disconnect, landscape
For social relations improvement	Social activity, local people, community-oriented

**Table 4:** Composition of study questionnaire.

Categories	Sub-Categories	Items
Demographic survey	-	13
Wellness tourism motivation	Relaxation/healing/escape from daily life	4
	Health improvement (mental and physical)	6
	Novelty	3
	Luxury and prestige	4
	Self-examination/education	4
	Nature friendliness	4
	Purpose of social relations improvement	4
Satisfaction	-	3
Revisit intention	-	3
Recommendation intention	-	3
Flow	-	3

**Table 5:** Result of Frequency Analysis.

Characteristic		N (%)	Characteristic		N (%)
Age	20~29	11 (4.3%)	Gender	Female	205 (79.8%)
	30~39	15 (5.8%)		Male	52 (20.2%)
	40~49	22 (8.6%)	Income	Less than KRW 1 million	27 (10.7%)
	50~59	100 (38.9%)		KRW 1 to 1.99 million	19 (7.5%)
	60~69	109 (42.4%)		KRW 2 to 2.99 million	42 (16.7%)
Education level	Below high school graduate	5 (1.9%)		KRW 3 to 3.99 million	50 (19.8%)
				KRW 4 to 4.99 million	42 (16.7%)
				Over KRW 5 million	72 (28.6%)
			Job	Public officer/Soldier	37 (14.3%)
	Graduated from high school	61 (23.6%)		Office worker	38 (14.7%)
				Technical worker	8 (3.1%)
	Graduated from university	127 (49.2%)		Student	5 (1.9%)
	Currently in graduate school or graduated from it	65 (25.2%)		Specialized job (professor, doctor, etc.)	19 (7.3%)
Marital status	Married	206 (80.2%)		Not employed/retired	36 (13.9%)
	Unmarried	51 (19.8%)			
				Housewife	67 (25.9%)
				Etc.	49 (18.9%)
Disease Status	Have a disease	110 (42.8%)	Type Of Product	A day type	97 (36.5%)
				Stay type	169 (63.5%)
	No disease	147(57.2%)			

**Table 6:** Results of factor analysis of measurement items for motivations for participation in wellness tourism products.

Variable	Measurement Item	Factor Loading	Eigenvalue	Variance Explanatory Power (%)	Reliability
Relaxation/healing/health improvement	Escape from boring everyday life	0.782	4.778	17.063	0.911
	Consolation for me, tired of repeated daily life	0.750			
	Physical function improvement	0.704			
	Stress relief	0.701			
	Mental function improvement	0.675			
	Psychological anxiety healing	0.664			
	Disease prevention and healing	0.653			
	Overall health maintenance/improvement	0.617			
	Leisure activities	0.498			
Self-examination/education	Confidence enhancement	0.831	3.725	13.301	0.919
	Self-esteem growth	0.824			
	Detailed recognition of the being called me	0.739			
	Detailed education on nature and wellness	0.531			
Nature friendliness	Feeling the beauty of nature	0.896	3.714	13.263	0.948
	Appreciation of natural scenery	0.877			
	Fresh air feeling	0.874			
	Communion with nature	0.812			
Luxury/prestige	Luxurious tourism experience	0.822	3.235	11.552	0.885
	Experience of tourism that treats tourists better and cares for them	0.763			
	Upgraded experience that could not be experienced by others	0.752			
	Being proud of a more upgraded experience	0.698			
Social relations improvement	Companionship maintenance/improvement	0.806	2.743	9.798	0.816
	Memories with companions	0.759			
	Interpersonal competency improvement	0.744			
	Securing confidence about strangers	0.671			
Novelty	Relief of curiosity about wellness tourism experience	0.824	2.674	9.549	0.877
	Unique tourism and wellness experience activities	0.794			
	New tourism different from existing one	0.781			

Total variance explanatory power: 74.528%; KMO: 0.892; Bartlett sphericity test: 6235.474 (*p* = 0.000).

**Table 7:** Results of factor analysis of overall satisfaction with wellness tourism product measurement items.

Variable	Measurement Item	Factor Loading	Eigenvalue	Variance Explanatory Power (%)	Reliability
Overall satisfaction	Overall satisfaction with wellness tourism	0.963	2.719	90.633	0.948
	Satisfaction with the wellness program	0.949			
	Satisfaction with the local (surrounding) environment where the wellness program was implemented	0.944			

Total variance explanatory power: 90.633%; KMO: 0.764,;Bartlett sphericity test: 780.175 (*p* = 0.000).

**Table 8:** Results of factor analysis of measurement items for intentions to revisit wellness tourism products.

Variable	Measurement Item	Factor Loading	Eigenvalue	Variance Explanatory Power (%)	Reliability
Revisit intention	Intention to participate in similar wellness programs (including those in other regions)	0.945	2.610	86.985	0.924
	Intention to participate in other wellness programs in the region	0.944			
	Intention to tour in the program implementation area (surroundings) again	0.909			

Total variance explanatory power: 86.985%; KMO: 0.748; Bartlett sphericity test: 630.693 (*p* = 0.000).

**Table 9:** Results of factor analysis of measurement items for intentions to recommend wellness tourism products.

Variable	Measurement Item	Factor Loading	Eigenvalue	Variance Explanatory Power (%)	Reliability
Recommendation intention	Intention to recommend others to participate in wellness-related activities and programs	0.984	2.843	94.774	0.972
	Intention to recommend wellness programs to others	0.981			
	Intention to recommend the wellness program area to others	0.955			

Total variance explanatory power: 94.774%; KMO: 0.744; Bartlett sphericity test: 1221.156 (*p* = 0.000).

**Table 10:** Results of factor analysis of wellness tourism product flow measurement items.

Variable	Measurement Item	Factor Loading	Eigenvalue	Variance Explanatory Power (%)	Reliability
Flow	I didn’t know that time was passing when I participated in the program.	0.956	2.616	87.206	0.926
	I completely flowed when I participated in the program.	0.935			
	I didn’t care about anything else when I participated in the program.	0.910			

Total variance explanatory power: 87.206%; KMO: 0.735; Bartlett sphericity test: 651.647 (*p* = 0.000).

**Table 11:** Verification of differences between clusters according to wellness tourism participation motivators.

Name	Cluster 1 n = 35 (13.5%)	Cluster 2 n = 89 (34.2%)	Cluster 3 n = 70 (26.9%)	Cluster 4 n = 66 (25.4%)	Means	F-Value	*p*
Relaxation/healing/health improvement	3.89	5.93	4.61	5.27	5.13	61.069	0.000
Novelty	5.21	6.15	4.36	5.97	5.48	80.125	0.000
Luxury/prestige	2.87	5.71	3.45	4.56	4.41	113.103	0.000
Self-examination/education	2.94	6.12	4.30	5.08	4.92	154.530	0.000
Nature friendliness	5.54	6.44	5.28	6.18	5.91	32.263	0.000
Social relations improvement	2.91	5.77	4.40	3.47	4.42	123.543	0.000
Mean	3.89	6.02	4.40	5.09	5.06	295.373	0.000
Cluster name	Novelty-seeking type	Comprehensive motivation-seeking type	Neutral wellness-seeking type	Exploratory wellness-seeking type	-	-	-

**Table 12:** Difference in the means of overall satisfaction, behavioral intention, and flow by wellness tourism product participant group.

Categories	Overall Satisfaction	Revisit Intention	Recommendation Intention	Flow	Means
Cluster 1 (Novelty-seeking type)	5.9143	5.9429	6.0952	5.7429	5.923825
Cluster 2 (Comprehensive motivation-seeking type)	6.2576	6.2689	6.3371	6.1798	6.26085
Cluster3 (Neutral wellness-seeking type)	5.7762	5.6952	5.7	5.2333	5.601175
Cluster4 (Exploratory wellness-seeking type)	6.2778	6.2323	6.2879	6.0707	6.217175
Means	6.0862	6.0605	6.1205	5.8385	
F-value	5.548 **	6.763 ***	7.067 ***	13.168 ***	

Significance probability: **: *p* < 0.010, ***: *p* < 0.001.

**Table 13:** Differences in the means of overall satisfaction among wellness tourism product participant groups.

Dependent Variable	Cluster Classification (I)	Cluster Analysis (J)	Mean Difference (I − J)	Standardized Error	Significance Probability	F-Value
Overall satisfaction	Novelty-seeking type (a) (m = 5.91)	b	−0.34329	0.17504	0.281	5.548 (*p* = 0.000)
		c	0.13810	0.18133	0.901	
		d	−0.36349	0.18315	0.271	
	Comprehensive motivation-seeking type (b) m = 6.26	a	0.34329	0.17504	0.281	
		c	0.48139 **	0.14028	0.009	
		d	−0.02020	0.14263	0.999	
	Neutral wellness-seeking type (c) m = 5.78	a	−0.13810	0.18133	0.901	
		b	−0.48139 **	0.14028	0.009	
		d	−0.50159 *	0.15028	0.012	
	Exploratory wellness-seeking type (d) m = 6.28	a	0.36349	0.18315	0.271	
		b	0.02020	0.14263	0.999	
		c	0.50159 *	0.15028	0.012	

Scheffe: b ≠ c/c ≠ d; * *p* < 0.05, ** *p* < 0.01.

**Table 14:** Differences in means of intention to revisit among wellness tourism product participant groups.

Dependent Variable	Cluster Classification (I)	Cluster Analysis (J)	Mean Difference (I − J)	Standardized Error	Significance Probability	F-Value
Revisit intention	Novelty-seeking type (a) (m = 5.94)	b	−0.32608	0.17521	0.328	6.763 (*p* = 0.000)
		c	0.24762	0.18150	0.602	
		d	−0.28947	0.18333	0.478	
	Comprehensive motivation-seeking type (b) m = 6.27	a	0.32608	0.17521	0.328	
		c	0.57370 **	0.14041	0.001	
		d	0.03662	0.14276	0.996	
	Neutral wellness-seeking type (c) m = 5.70	a	−0.24762	0.18150	0.602	
		b	−0.57370 **	0.14041	0.001	
		d	−0.53709 **	0.15043	0.006	
	Exploratory wellness-seeking type (d) m = 6.23	a	0.28947	0.18333	0.478	
		b	−0.03662	0.14276	0.996	
		c	0.53709 **	0.15043	0.006	

Scheffe: b ≠ c/c ≠ d; ** *p* < 0.01.

**Table 15:** Differences in means of recommendation intention among wellness tourism product participant groups.

Dependent Variable	Cluster Classification (I)	Cluster Analysis (J)	Mean Difference (I − J)	Standardized Error	Significance Probability	F-Value
Recommendation intention	Novelty-seeking type (a) (m = 5.94)	b	−0.24184	0.18599	0.640	7.067 (*p* = 0.000)
		c	0.39524	0.19299	0.244	
		d	−0.19264	0.19493	0.807	
	Comprehensive motivation-seeking type (b) m = 6.27	a	0.24184	0.18599	0.640	
		c	0.63708 **	0.14892	0.001	
		d	0.04920	0.15143	0.991	
	Neutral wellness-seeking type (c) m = 5.70	a	−0.39524	0.19299	0.244	
		b	−0.63708 **	0.14892	0.001	
		d	−0.58788 **	0.15994	0.004	
	Exploratory wellness-seeking type (d) m = 6.23	a	0.19264	0.19493	0.807	
		b	−0.04920	0.15143	0.991	
		c	0.58788 **	0.15994	0.004	

Scheffe: b ≠ c/c ≠ d; ** *p* < 0.01.

**Table 16:** Differences in means of flow among wellness tourism product participant groups.

Dependent Variable	Cluster Classification (I)	Cluster Analysis (J)	Mean Difference (I − J)	Standardized Error	Significance Probability	F-Value
Flow	Novelty-seeking type (a) (m = 5.74)	b	−0.43692	0.20047	0.194	13.168 (*p* = 0.000)
		c	0.50952	0.20801	0.114	
		d	−0.32785	0.21010	0.488	
	Comprehensive motivation-seeking type (b) m = 6.17	a	0.43692	0.20047	0.194	
		c	0.94644 ***	0.16052	0.000	
		d	0.10907	0.16322	0.930	
	Neutral wellness-seeking type (c) m = 5.23	a	−0.50952	0.20801	0.114	
		b	−0.94644 ***	0.16052	0.000	
		d	−0.83737 ***	0.17239	0.000	
	Exploratory wellness-seeking type (d) m = 6.07	a	0.32785	0.21010	0.488	
		b	−0.10907	0.16322	0.930	
		c	0.83737 ***	0.17239	0.000	

Scheffe: b ≠ c/c ≠ d; *** *p* < 0.001.

## Data Availability

Not applicable.
